# Successful management of temporary veno-venous extracorporeal membrane oxygenation for a pediatric lung transplant recipient with bronchiolitis obliterans syndrome awaiting lung re-transplantation: a case report

**DOI:** 10.1186/s40792-023-01742-4

**Published:** 2023-09-15

**Authors:** Yasuaki Tomioka, Kentaroh Miyoshi, Shin Tanaka, Seiichiro Sugimoto, Rie Kanai, Tetsuro Nikai, Shinichi Toyooka, Masaomi Yamane

**Affiliations:** 1 Division of Thoracic Surgery, Department of Surgery, Faculty of Medicine, Shimane University, 89-1 Enya-Cho, Izumo, Shimane 693-8501 Japan; 2 Department of General Thoracic Surgery and Breast and Endocrinological Surgery, Okayama University Graduate School of Medicine, Dentistry and Pharmaceutical Sciences, Okayama, Japan; 3 Department of General Thoracic Surgery and Organ Transplant Center, Okayama University Hospital, Okayama, Japan; 4 Department of Pediatrics, Faculty of Medicine, Shimane University, Izumo, Japan; 5 Department of Anesthesiology, Faculty of Medicine, Shimane University, Izumo, Shimane Japan

**Keywords:** Lung transplantation, ECMO, Tracheostomy, Rehabilitation, Re-transplantation, Case report

## Abstract

**Background:**

The use of extracorporeal membrane oxygenation (ECMO) as a bridge to lung transplantation is an uncommon strategy in Japan owing to the severe donor shortage and absence of urgent allocation policy. Moreover, the use of veno-venous (VV) ECMO for immunosuppressed patients is controversial; thus, applying ECMO to patients who await lung re-transplantation is challenging.

**Case presentation:**

A 16-year-old lung transplant recipient with grade 3 bronchiolitis obliterans syndrome was waitlisted for lung re-transplantation. Eleven months later, he fell into severe respiratory acidosis with hypercapnia, which were not resolved with mechanical ventilation. VV ECMO was introduced to minimize lung stress and strain. Tracheostomy was additionally performed on day 5 after the start of ECMO, and respiratory condition swiftly improved; hence, the weaning process from VV ECMO began on day 9. Rehabilitation became implementable, and bilateral re-lung transplantation was successfully performed 6 months after the ECMO treatment. No critical complication related to the precedent use of ECMO was noted.

**Conclusions:**

VV ECMO can be a feasible treatment option even for lung transplant candidates awaiting re-transplantation for a prolonged period. Introduction of ECMO and tracheostomy in the early deterioration stage may be crucial to successful subsequent patient management.

## Background

The number of brain-dead organ donations in Japan has increased in recent years; however, it remains insufficient to meet the demands. There remains a shortage of suitable lung donors, especially for pediatric patients or candidates for re-transplantation [1]. In Japan, wait-listing for lung transplantation (LT) is based on the order of registration with no established system to prioritize urgent patients. Thus, waiting time for each candidate cannot be estimated, and the use of extracorporeal membrane oxygenation (ECMO) as a bridge to LT is considered controversial and uncommon. In addition, for the indication of ECMO, its use in immunosuppressed or severely immunocompromised patients has been considered a relative contraindication [2]. That is, respiratory failure associated with hematopoietic stem cell transplantation (HSCT) or chronic rejection after LT is a controversial indication for ECMO. Overall, determining whether ECMO should be applied to wait-listed patients with history of those transplantation demands a challenging decision-making.

Herein, we present a case of management of veno-venous (VV) ECMO for a pediatric lung transplant recipient with end-stage bronchiolitis obliterans syndrome (BOS) who awaited lung re-transplantation. The patient was weaned from VV ECMO after tracheostomy and ECMO management to remove CO_2_ within a short period. Thereafter, adequate rehabilitation was successfully implemented to survive waiting time for lung re-transplantation.

## Case presentation

A 10-year-old boy (height 123.8 cm, weight 24.9 kg) developed irreversible lung failure associated with chronic graft-versus-host disease following HSCT. He primarily underwent right single living-donor lobar LT donated from the next of kin followed by long-term immunosuppressive therapy. At 14 years old, the patient developed chronic lung allograft dysfunction (CLAD, BOS) with concomitant development of de novo donor-specific alloantibody (DSA) toward DQ4 (16,880 mean fluorescence intensity; MFI), which resulted in a rapid decline in respiratory function to grade 3 or < 50% of baseline forced expiratory volume in 1 s of 0.46 L (19.3% of predicted value). The patient was suspected of having a clinical antibody-mediated rejection. Shortly after the diagnosis of CLAD (BOS), he was registered and waitlisted for lung re-transplantation from a deceased donor in the Japan Organ Transplantation Network. Antigen-targeted therapy (plasma exchange: 4 times) was conducted but irresponsive (MFI: 11453 → 2483 → 14834). Owing to progression of respiratory failure, the patient was hospitalized and prescribed non-invasive positive pressure ventilation 7 months after registration on the list. During the early waiting period, the patient was relatively in stable conditions (pCO2, 60 Torr), maintaining moderate physical performance to implement daily rehabilitation program of ambulation and cycle ergometry.

**Fig. 1:**
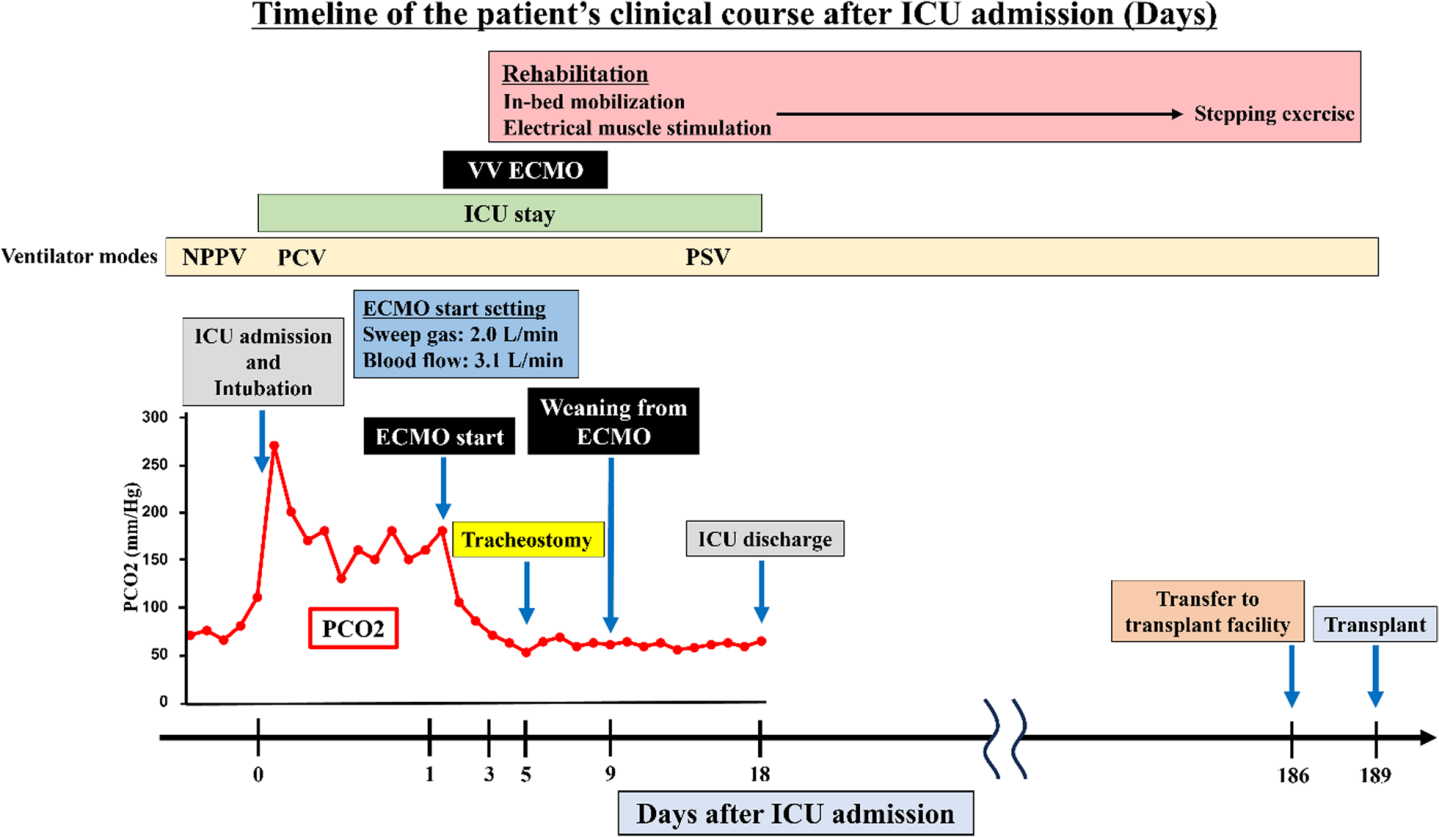
Timeline of the patient’s clinical course after ICU admission. *ECMO* extracorporeal membrane oxygenation, *ICU* intensive care unit, *NPPV* non-invasive positive pressure ventilation, *PCO2* partial pressure of carbon dioxide, *PCV* pressure control ventilation, *PSV* pressure support ventilation, *VV ECMO* veno-venous extracorporeal membrane oxygenation

**Fig. 2:**
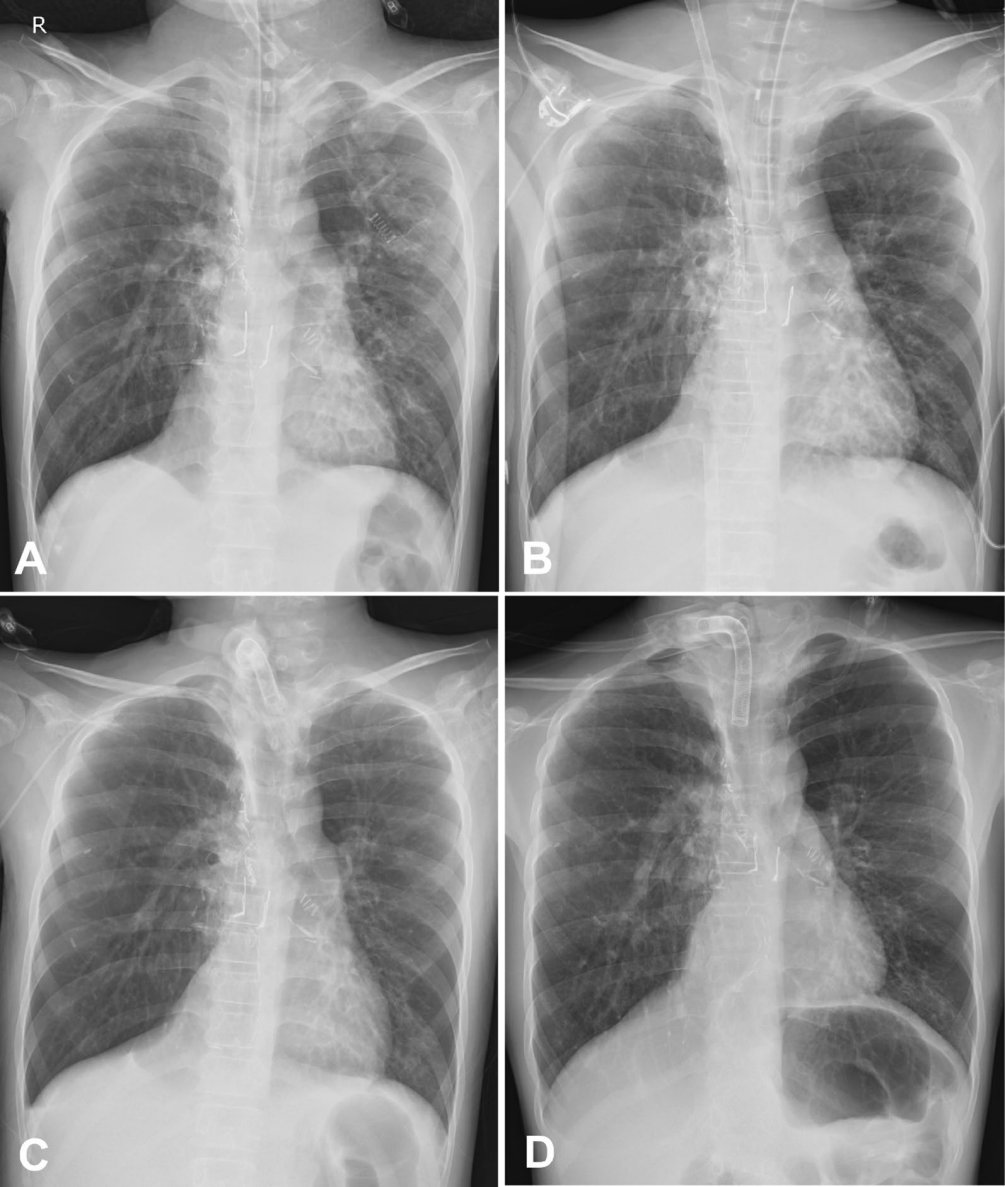
Chest X-ray images during the patient’s clinical course: **A** day 0 after ICU admission; **B** after ECMO initiation; **C** after removing ECMO; **D** before transfer. *ECMO* extracorporeal membrane oxygenation, *ICU* intensive care unit

However, his respiratory condition rapidly deteriorated 11 months after wait-listing (the age of 15), and he (height 143 cm, weight 33 kg) was admitted to the intensive care unit (ICU) and required mechanical ventilation under sedation because of hypercapnic respiratory failure (Fig. 1). Despite the high level of positive end-expiratory pressure, and prolonged expiratory time, the patient’s severe respiratory acidosis (pH, 6.9–7.1; pCO2, 130–200 Torr) with hypercapnia did not improve, and an alternate mode of lung support was required as rescue therapy (Fig. 2A). The patient had chronic deterioration of respiratory function; hence, he possibly would not be weaned off VV ECMO once initiated. However, the patient remained free from active bacterial, viral, or fungal infection, and the function of other vital organs including the heart, kidney, and liver was normal. Following emergency multidisciplinary team discussions, VV ECMO was introduced initially as a bridging strategy to transplantation on day 1 after ICU admission, with the aim to achieve long-term standby status for LT (Fig. 2B). A 21-Fr single catheter was cannulated for venous drainage in the common femoral vein and a 19-Fr catheter for return in the right internal jugular vein. We had planned to switch to cannulation with a single-site dual-lumen device if the patient transitioned to a state where rehabilitation was possible under a prolonged period of ECMO use. The initial ECMO settings were as follows: sweep gas flow, 2.0 L/min; flow, 3.1 L/min; 3360 revolutions per min (RPM); and heparin infusion. ECMO and ventilator settings were later adjusted to minimize lung stress and strain. Early rehabilitation of in-bed mobilization and electrical muscle stimulation was started on day 2 after ECMO initiation. Subsequently, a tracheostomy was placed on day 5. Once the blood gas data were normalized, the weaning process from VV ECMO was attempted on day 6. Following the cautious reduction of the flow and several weaning trials, the ECMO could finally be removed on day 9 (Fig. 2C) and sedation was completely ceased on day 18. The patient underwent rehabilitation under ventilator support, and his physical performance improved to the extent that he could perform stepping exercise.

Six months after the ECMO treatment, a suitable organ was allocated. The patient was transferred to the referred lung transplant center (Fig. 2D), and bilateral LT was performed perioperatively using desensitization therapy (DSA DQ4 [MFI:13529]). Intraoperative cardiopulmonary support was required, but could be removed immediately after implantation. Standard triple immunosuppressive therapy (tacrolimus, mycophenolate mofetil, and prednisolone), and antiviral and antifungal prophylaxis were administered postoperatively. No major surgical complication related to the previous history of ECMO use, including severe primary graft dysfunction, cardiac insufficiency, infection, and venous thrombosis, was noted. The patient was successfully weaned off mechanical ventilation on postoperative day 69 and oxygen inhalation on day 74. He was transferred to our rehabilitation facility on postoperative day 99 and was finally discharged on day 114. He returned to his normal daily life without any significant complications 14 months after lung re-transplantation.

## Discussion

There is currently an increasing number of LT recipients who require lung re-transplantation because of advanced chronic lung allograft dysfunction. We report a case of respiratory failure in a pediatric LT recipient with high-grade BOS that was managed with short-term VV ECMO, resulting in successful wait-list survival for re-transplantation. Since there is a wide variety of patient conditions where use of ECMO is considered, it is challenging to standardize its indication and management [2]. The current case provides an implication regarding patient selection for ECMO use in those specific situations and pre- and post-ECMO management.

Lung transplant candidates are generally in irreversible respiratory failure due to progressive lung disease; hence, weaning these patients from ECMO can be challenging. As the average waiting time for deceased donor LT is > 800 days in Japan [1]. There is some case anecdote reported regarding successful management of extremely long-term bridging ECMO before LT [3], whereas decision-making for bridging ECMO is always difficult. In addition, evidence suggests that the use of ECMO in immunocompromised patients with respiratory failure increases hospital mortality; thus, the introduction of ECMO after HSCT or in an immunosuppressed condition remains controversial [2, 4]. In this case, the patient had a history of HSCT, living-donor LT, and long-term treatment with immunosuppressive therapy. Therefore, the decision to introduce ECMO was carefully made with various factors considered, including physical performance, absence of active infection or non-lung organ damage, and availability of a medical service that allowed rehabilitation while on ECMO. Furthermore, the clinical course of respiratory deterioration was acute on chronic progression, and the patient’s physical activity was maintained until a few months before the event. We expected that he could survive if the acute element of respiratory deterioration would be resolved. All of these conditions considered, we decided to introduce ECMO.

Regarding patient management, prolonged pre-ECMO invasive mechanical ventilation (> 7 days) is reportedly associated with increased mortality [5–8]. In fact, exceeding 7 days from intubation to ECMO is crucial in well-established risk prediction scores and is often considered a relative contraindication for ECMO therapy [5, 6, 9–12]. Therefore, the application of ECMO should be immediately discussed among relevant medical professionals once the criteria are met. In the current case, the patient had already developed severe hypercapnic respiratory failure due to respiratory muscle fatigue prior to ICU admission, which could not be resolved by mechanical ventilation support alone. Prolonged exposure to hypercapnic respiratory failure and the accompanying acidosis can have detrimental effects on other organs and complicate the subsequent recovery process. The early initiation of ECMO is believed to have interrupted such a vicious cycle, leading to avoidance of fatal side effects and successful weaning from ECMO. In addition, recent reports have revealed that early tracheostomy after VV ECMO initiation is associated with shorter ECMO support duration and ICU stay and lower hospital mortality rates [13, 14]. In this case, the patient underwent tracheostomy on day 5 after VV ECMO initiation and was subsequently weaned off VV ECMO within a short period. Converting airway management from oral intubation to tracheostomy tube allows for reduction of airway dead space and resistance, aggressive patient’s mobilization, chest physiotherapy, and a more straightforward secretion clearance [13]. Diligent rehabilitation during bridging ECMO is associated with positive outcomes after LT [15]. Ambulation after tracheostomy improves strength and pulmonary mechanics and facilitates lung recovery and weaning from ECMO support [13]. Overall, early ECMO initiation to flush retained CO_2_ out, early tracheostomy, and implementation of thorough rehabilitation comprehensively contributed to the favorable outcome.

## Conclusions

VV ECMO can be a feasible treatment option in case of respiratory deterioration of LT candidates on the long waiting list if careful consideration is given before application. Early judgment of treatment escalation in each step is key to successful management of patients requiring ECMO in this specific adverse situation. However, for the patient with multiple high-risk factors, long-term follow-up is essential to conclusively determine the appropriateness of treatment.

## Data Availability

Not applicable.

## Abbreviations

BOS: Bronchiolitis obliterans syndrome
CLAD: Chronic lung allograft dysfunction
DSA: Donor-specific antibody
ECMO: Extracorporeal membrane oxygenation
HSCT: Hematopoietic stem cell transplantation
ICU: Intensive care unit
MFI: Mean fluorescence intensity
LT: Lung transplantation
VV: Veno-venous
